# Analysis of the Thermally Induced Packaging Effects on the Frequency Drift of Micro-Electromechanical System Resonant Accelerometer

**DOI:** 10.3390/mi14081556

**Published:** 2023-08-03

**Authors:** Xiaorui Bie, Xingyin Xiong, Zheng Wang, Wuhao Yang, Zhitian Li, Xudong Zou

**Affiliations:** 1 Aerospace Information Research Institute, Chinese Academy of Sciences, Beijing 100190, China; biexr@aircas.ac.cn (X.B.); xiongxy@aircas.ac.cn (X.X.); yangwh@aircas.ac.cn (W.Y.); lizt@aircas.ac.cn (Z.L.); 2 Shangdong Key Laboratory of Low-Altitude Airspace Surveillance Network Technology, QiLu Aerospace Information Research Institute, Jinan 250101, China; wangzheng02@aircas.ac.cn

**Keywords:** MEMS resonant accelerometer, frequency drift, packaging effects, thermo-mechanical stress, finite element method

## Abstract

Due to the working principle of MEMS resonant accelerometers, their thermally induced frequency drift is an inevitable practical issue for their extensive application. This paper is focused on reducing the thermally induced packaging effects on the frequency drift. A leadless ceramic chip carrier package with a stress-buffering layer was proposed for a MEMS resonant accelerometer, and the influences of packaging structure parameters on the frequency drift were investigated through finite element simulations and verified experimentally. Because of the thermal mismatch between dissimilar materials, the thermo-mechanical stress within the resonant beam leads to a change in the effective stiffness and causes the frequency drift to decrease linearly with increasing temperature. Furthermore, our investigations reveal that increasing the stress-buffering layer thickness and reducing the solder layer thickness can significantly minimize the thermo-mechanical stress within the resonant beam. As the neutral plane approaches the horizontal symmetry plane of the resonant beam when optimizing the packaging structure, the effects of the compressive and tensile stresses on the effective stiffness of the resonant beam will cancel each other out, which can dramatically reduce the frequency drift. These findings provide guidelines for packaging design through which to improve the temperature stability of MEMS resonant accelerometers.

## 1. Introduction

A micro-electromechanical system (MEMS) resonant accelerometer is a kind of micro-inertial device that converts external acceleration into resonant frequency variations of the resonant beam. It not only has the advantages of other MEMS devices, like small size, light weight, low power consumption, and low cost, but also has the characteristics of resonant devices, such as quasi-digital output, strong anti-jamming capability, high measurement accuracy, and excellent stability. In recent years, MEMS resonant accelerometers have become a research hotspot for relevant scholars, and they have been widely used in high-precision inertial navigation systems [1,2,3].

Ideally, the resonant frequency of the resonant beam in a MEMS resonant accelerometer is only sensitive to external acceleration. However, thermally induced frequency drift is an inevitable practical issue that impacts the scale factor and zero-bias stability of a MEMS resonant accelerometer [4,5]. The thermal effects on the frequency drift of MEMS resonant accelerometers mainly include changes in the structural dimensions of the resonant beam and the Young’s modulus of monocrystalline silicon, caused by temperature variation [3,6]; temperature-dependent intrinsic residual stress within the resonant beam, produced during the manufacturing process [7,8]; and thermo-mechanical stress generated during the packaging process, due to the thermal mismatch between dissimilar materials [9,10]. Therefore, how to reduce the thermally induced frequency drift is the crucial problem to be solved urgently. Many efforts have been focused on significantly enhancing the scale factor and zero-bias stability of MEMS resonant accelerometers, such as novel temperature-insensitive device structure design [11,12]; stress isolation, with which to minimize the thermo-mechanical stress transmitted into the device [13]; operating temperature controls for the micro-oven integrated in the device [14]; and electronic temperature compensation of the output signal, either through the hardware or software system [15,16]. In addition, packaging material selection and structure optimization are also effective ways to radically reduce the thermal effects on MEMS resonant accelerometers [17,18,19], which, meanwhile, do not increase the power consumption or complexity of the system.

This paper is primarily focused on the frequency drift of MEMS resonant accelerometers caused by thermo-mechanical stress, which is attributed to the coefficient of thermal expansion (CTE) mismatch between dissimilar materials in the package, referred to as the thermally induced packaging effects [20]. Firstly, the working principle of the MEMS resonant accelerometer was presented, and the mechanism of thermally induced frequency drift was analyzed. Then, a leadless ceramic chip carrier (LCCC) package, with a stress-buffering layer, was proposed for the MEMS resonant accelerometer, and the MEMS die was soldered onto the LCCC socket with eutectic lead-free solder alloy. In order to clarify the influences of packaging structure parameters on the thermo-mechanical stress within the MEMS resonant accelerometer, thermo-mechanical simulations of the die attachment process were conducted using the finite element method (FEM). After that, the resonant frequencies of the resonant beam were obtained via prestressed modal simulations. Finally, the simulation results were experimentally verified via temperature tests for the packaged MEMS resonant accelerometer. The findings showed that increasing the stress-buffering layer thickness and reducing the solder layer thickness can significantly minimize the thermo-mechanical stress within the resonant beam. As the neutral plane approaches the horizontal symmetry plane of the resonant beam, the effects of thermo-mechanical stress on the effective stiffness of the resonant beam become dramatically reduced, along with the frequency drift. This paper explores an effective method of thermo-mechanical stress control during the packaging process, which can provide guidelines for packaging design through which to improve the temperature stability of MEMS resonant accelerometers.

## 2. Working Principle of MEMS Resonant Accelerometer and Frequency Drift Analysis

### 2.1. Working Principle of MEMS Resonant Accelerometer

**Figure 1:**
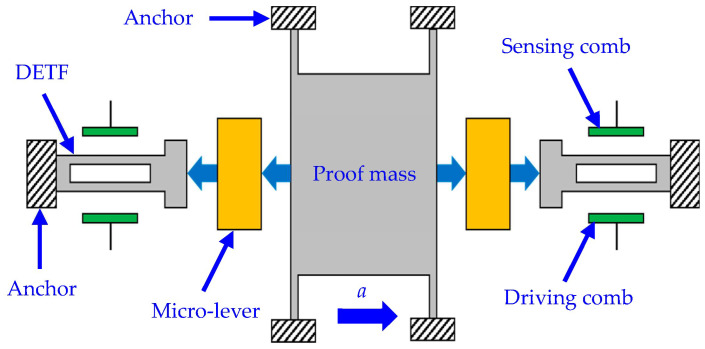
Schematic diagram of a MEMS resonant accelerometer.

A MEMS resonant accelerometer is a force-sensitive sensor that detects external acceleration according to the force–frequency characteristics of the resonant beam. A schematic diagram of a MEMS resonant accelerometer is shown in Figure 1, consisting of double-ended turning forks (DETFs), a proof mass, micro-levers, combs, and anchors [21]. The DETFs work as force-sensitive resonant beams, one end of which is fixed to the anchors and the other end of which is connected to the proof mass, which converts external acceleration into inertial force. The generated inertial force is magnified by the micro-levers, subsequently transmitted into the DETFs along the sensitive axes of the resonant beams, and subjects the DEFTs to tensile or compressive force. Therefore, the resonant frequency of the DEFT under tensile force will increase, whereas that of the DEFT under compression force will decrease. The magnitude of the external acceleration can be derived from the detected resonant frequency variations.

Without considering the axial force effects, the resonant frequency of DETFs is obtained via the following equation [4,22]:

(1) $$f_{0}=\frac{1}{2\pi }\sqrt{\frac{K_{\mathrm{eff}}}{M_{\mathrm{eff}}}}$$

where *M*_eff_ is the effective mass of the resonant beam and *K*_eff_ is the effective stiffness of the resonant beam, which is obtained via the following equation:

(2) $$K_{\mathrm{eff}}=A\frac{E_{\mathrm{Si}}h}{12}{\left(\frac{w}{l}\right)}^{3}$$

where *A* is the constant related to the vibration mode of the resonant beam; *l*, *w*, and *h* are the length, width, and thickness of the resonant beam, respectively; and *E*_Si_ is the Young’s modulus of monocrystalline silicon. Accordingly, Equation (1) can be expressed as

(3) $$f_{0}=\frac{1}{2\pi }\sqrt{A\frac{E_{\mathrm{Si}}h}{12}{\left(\frac{w}{l}\right)}^{3}/M_{\mathrm{eff}}}$$

While the axial force is loaded to the resonant beams, the effective stiffness of the DETFs is obtained via the following equation [4]:

(4) $$K_{\mathrm{eff}}\left(F\right)=A\frac{E_{\mathrm{Si}}h}{12}{\left(\frac{w}{l}\right)}^{3}+B\frac{F}{l}$$

where *B* is the constant related to the vibration mode of the resonant beam and *F* is the axial force, which may be tensile (*F* > 0) or compressive (*F* < 0). Hence, the resonant frequency of axially loaded DETFs can be expressed as

(5) $$f\left(F\right)=\frac{1}{2\pi }\sqrt{\left[A\frac{E_{\mathrm{Si}}w}{12}{\left(\frac{h}{l}\right)}^{3}+B\frac{F}{l}\right]/M_{\mathrm{eff}}}$$

Rearranging Equation (5) gives

(6) $$f\left(F\right)=f_{0}\sqrt{1+C\frac{l^{2}}{E_{\mathrm{Si}}hw^{3}}F}$$

where *f*_0_ is the unloaded resonant frequency of DETFs and *C* is the constant related to the vibration mode of the resonant beam. Equation (6) indicates that the resonant frequency versus extrinsic inertial force relationship in a MEMS resonant accelerometer is nonlinear.

In order to determine the linear and higher-order scale factors of a MEMS resonant accelerometer, a Taylor series expansion of Equation (6) at the point *F* = 0 is constructed as follows [23]:

(7) $$f\left(F\right)=f_{0}\left(1+\frac{1}{2}pF-\frac{1}{8}p^{2}F^{2}+\frac{1}{16}p^{3}F^{3}-\cdots \right)$$

where *p* = *Cl*^2^/*E*_Si_*hw*^3^.

Equation (7) can be transformed into

(8) $$f\left(a\right)=f_{0}\left(1+K_{1}a+K_{2}a^{2}+K_{3}a^{3}+\cdots \right)$$

where *a* is the external acceleration, *K_n_* = *q_n_K*_1_*^n^*, *q_n_* = *q_n_*_−1_(3 − 2*n*)/*n*, *K*_1_ = *p*/2, and *q*_1_ = 1. In practical applications, because the sensing range of a MEMS resonant accelerometer lies in the linear region of the scale factor, the higher-order nonlinear terms in Equation (8) can be ignored. Therefore, the resonant frequency versus external acceleration relationship can be rewritten as

(9) $$f\left(a\right)\approx f_{0}\left(1+C\frac{l^{2}}{2E_{\mathrm{Si}}hw^{3}}a\right)$$

By means of Equation (9), the external acceleration can be calculated from the detected resonant frequency signal of a MEMS resonant accelerometer.

### 2.2. Thermal Effects on the Frequency Drift of MEMS Resonant Accelerometer

From Equations (3) and (9), it can be seen that the unloaded resonant frequency and scale factor are dependent on the Young’s modulus of monocrystalline silicon and the dimensions of the resonant beam, which indicates that the temperature stability of a MEMS resonant accelerometer is correlated with the Young’s modulus sensitivity to temperature and the thermal expansion.

It is found that the Young’s modulus of monocrystalline silicon decreases linearly with increasing temperature [24], which can be expressed as

(10) $$E_{\mathrm{Si}}\left(T\right)=E_{\mathrm{Si}}{}_{0}\left[1-k_{E}\left(T-T_{0}\right)\right]$$

where *E*_Si0_ is the Young’s modulus of monocrystalline silicon at a temperature of *T*_0_; *T*_0_ usually is assumed to be room temperature; and *k_E_* is the temperature coefficient of the Young’s modulus, about 60 ppm/°C. Accordingly, the frequency drift of a MEMS resonant accelerometer induced by the Young’s modulus sensitivity to temperature can be described as

(11) $${\left[\frac{1}{f_{0}}\frac{\partial f_{0}}{\partial T}\right]}_{E}=\frac{1}{f_{0}}\frac{\partial f_{0}}{\partial E_{\mathrm{Si}}}\frac{\partial E_{\mathrm{Si}}}{\partial T}=-\frac{1}{2}k_{E}=-30\;\mathrm{ppm}/{}^{\circ}C$$

Moreover, the thermal expansion of monocrystalline silicon will lead to dimensional variations in the resonant beam as the temperature changes and, consequently, induce changes in the effective stiffness and scale factor of a MEMS resonant accelerometer. The frequency drift induced by the thermal expansion of monocrystalline silicon can be described as

(12) $${\left[\frac{1}{f_{0}}\frac{\partial f_{0}}{\partial T}\right]}_{\mathrm{CTE}}=\frac{1}{f_{0}}\left[{\left(\frac{\partial f_{0}}{\partial T}\right)}_{l}+{\left(\frac{\partial f_{0}}{\partial T}\right)}_{w}+{\left(\frac{\partial f_{0}}{\partial T}\right)}_{h}\right]=\frac{1}{2}\alpha_{\mathrm{Si}}=1.3\;\mathrm{ppm}/{}^{\circ}C$$

where *α*_Si_ is the linear CTE of monocrystalline silicon, about 2.6 ppm/°C at room temperature. It is found that the frequency drift of a MEMS resonant accelerometer is largely controlled by the Young’s modulus sensitivity to temperature, since the temperature coefficient of the resonant frequency is an order of magnitude larger than that correlated with the thermal expansion of monocrystalline silicon.

Apart from the Young’s modulus sensitivity to temperature and the thermal expansion of monocrystalline silicon, the resonant frequency is also impacted by the axial stress within the resonant beam. As indicated by Equation (6), the axial stress will lead to the zero drift of the MEMS resonant accelerometer, even without extrinsic inertial force. The axial stress effects not only come from the intrinsic residual stress produced during the manufacturing process but also the thermo-mechanical stress due to the thermal mismatch between dissimilar materials during the packaging process. In general, it is hard to physically model the real residual stress, and the residual stress at the wafer level plays a dominant role in a MEMS resonant accelerometer, which can be released by wafer dicing [7,25]. Hence, only the thermo-mechanical stress formed in chip-level packaging is taken into account, which is derived from the deformation inconsistency induced by the CTE mismatch between dissimilar materials, as well as the temperature gradient existing in the package.

This paper assumes a uniformly distributed temperature field in a MEMS resonant accelerometer, and when it drops to room temperature from curing temperature or soldering temperature, the thermo-mechanical stress induced by the CTE mismatch between the MEMS die and the ceramic substrate can be approximatively expressed as [26]

(13) $$\sigma =\frac{E_{\mathrm{Si}}}{1-v_{\mathrm{Si}}}\left|\Delta T\left(\alpha_{\mathrm{Si}}-\alpha_{\mathrm{sub}}\right)\right|$$

where *α*_sub_ is the linear CTE of the ceramic substrate, which is about 6.5 ppm/°C at room temperature for alumina ceramic; *v*_Si_ is the Poisson’s ratio of monocrystalline silicon, about 0.28 at room temperature; and Δ*T* is the temperature variation. By combining Equations (6) and (13), the frequency drift induced by the thermo-mechanical stress can be described as

(14) $$\left|\frac{1}{f_{0}}\frac{\partial f\left(\sigma \right)}{\partial T}\right|=\left|\frac{1}{f_{0}}\frac{\partial f\left(\sigma \right)}{\partial \sigma }\frac{\partial \sigma }{\partial T}\right|=\frac{C}{2}\frac{\left|\alpha_{\mathrm{Si}}-\alpha_{\mathrm{sub}}\right|}{1-v_{\mathrm{Si}}}{\left(\frac{l}{w}\right)}^{2}\frac{f_{0}}{f\left(\sigma \right)}$$

From Equation (14), it can be inferred that the magnitude of the temperature coefficient of resonant frequency correlated with the thermo-mechanical stress is comparable to that correlated with the Young’s modulus sensitivity to temperature and the thermal expansion of monocrystalline silicon, since the length-width ratio of the resonant beam is *l*/*w* >> 1. In other words, the thermo-mechanical stress formed in chip-level packaging also plays a dominant role in the frequency drift of a MEMS resonant accelerometer.

In summary, the thermal effects on the frequency drift of a MEMS resonant accelerometer are primarily dependent on the change in the Young’s modulus of monocrystalline silicon and the thermo-mechanical stress formed in chip-level packaging, rather than the thermal deformation of the resonant beam.

## 3. MEMS Resonant Accelerometer Device and Package

### 3.1. MEMS Resonant Accelerometer Device

**Figure 2:**
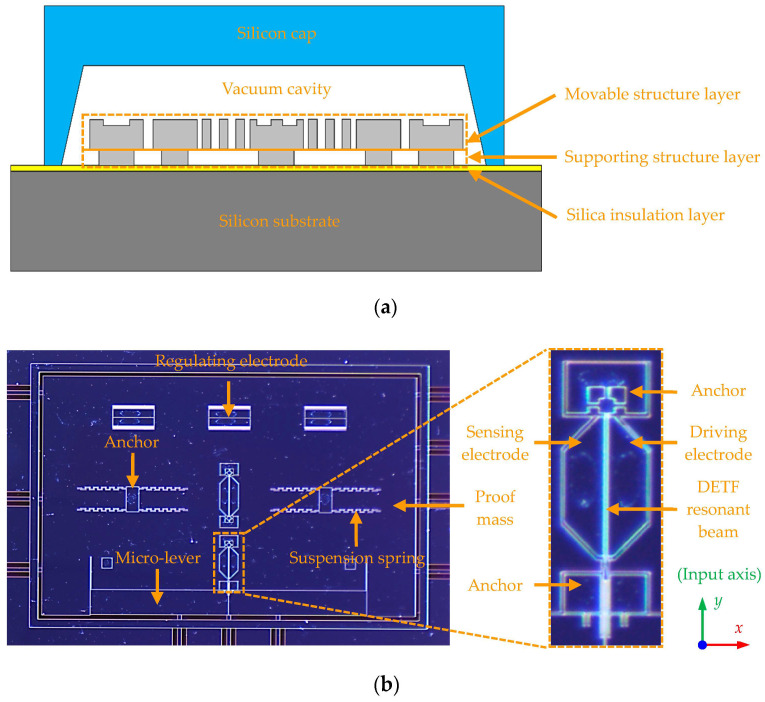
MEMS resonant accelerometer device fabricated for this study: (**a**) schematic cross-sectional view; (**b**) optical image of the movable structure layer.

Figure 2a shows a schematic cross-sectional view of the MEMS resonant accelerometer device fabricated for this study, which is a five-layered structure composed of the silicon substrate, the silica insulation layer, the supporting structure layer, the movable structure layer, and the silicon cap, from bottom to top. Figure 2b shows an optical image of the movable structure layer, consisting of the DETF resonant beam working as the force-frequency converter, the micro-levers serving as the force amplifiers, the proof mass generating the inertial force, the suspension springs suspending the proof mass, the anchors fixing the movable structures, and the electrodes with different functions.

The MEMS resonant accelerometer device was manufactured using the silicon-on-insulator (SOI) technique. The thickness of the 6-inch SOI wafer is 400 μm, including a 50 μm thick top silicon layer and a 2 μm thick silica insulation layer. Photolithography and a deep reactive ion etching (DRIE) process were successively conducted to define the supporting and movable structures on the top silicon layer of the SOI wafer. To protect the fabricated MEMS structures and ensure the hermeticity, a silicon cap wafer was subsequently placed over the SOI wafer through the glass frit bonding process in a vacuum chamber. Finally, the capped wafer was diced into singulated MEMS dies. The static performance tests of the MEMS resonant accelerometer device were performed at room temperature. The resonant frequency and Q-factor of the tested MEMS dies were evaluated to be approximately 180 kHz and 30,000, respectively. Furthermore, the sensing range of the tested MEMS dies is ±20 g and the scale factor is measured to be about 780 Hz/g.

### 3.2. MEMS Resonant Accelerometer Package

**Figure 3:**
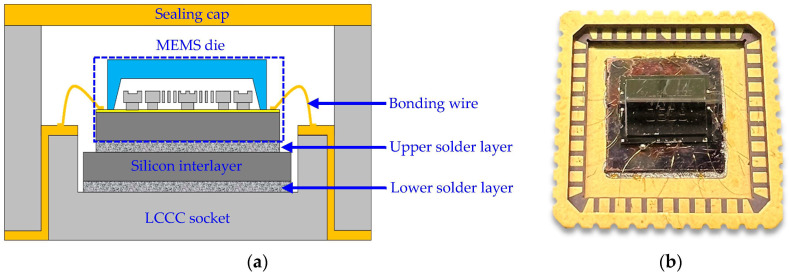
MEMS resonant accelerometer package designed for this study: (**a**) schematic cross-sectional view; (**b**) optical image of the assembled component without sealing cap.

Figure 3a shows a schematic cross-sectional view of the MEMS resonant accelerometer package designed for this study, including the MEMS die, LCCC socket, sealing cap, silicon interlayer, solder layers, and bonding wires. The MEMS die was encapsulated using the LCCC packaging technique and a silicon interlayer was adopted to connect the MEMS die and the LCCC socket, serving as the stress-buffering layer to relax the thermo-mechanical stress from the ceramic substrate. The MEMS die was soldered onto the LCCC socket with eutectic lead-free solder alloy instead of die attachment film (DAF) because of the strongly temperature-dependent material properties of DAF, which result in significant frequency drift of a MEMS resonant accelerometer as the temperature changes [19]. Figure 3b shows an optical image of the assembled components without sealing cap.

**Figure 4:**
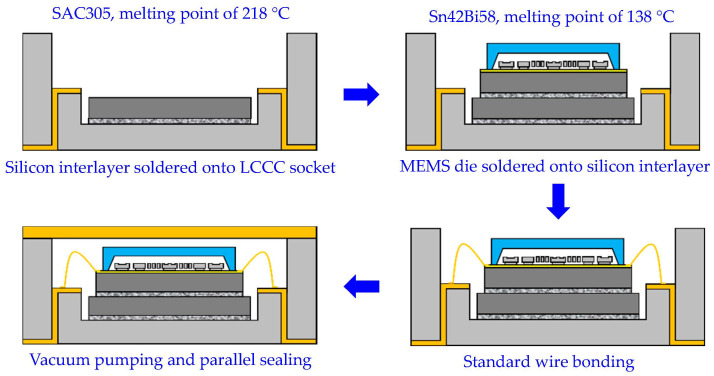
Packaging process of the MEMS resonant accelerometer.

The detailed packaging process of the MEMS resonant accelerometer is shown in Figure 4. Firstly, the silicon interlayer with double-sided metallization was soldered onto the LCCC socket using Sn3.0Ag0.5Cu (SAC305) solder alloy with a melting point of 218 °C, which was implemented through the reflow soldering process in a vacuum reflow oven, and followed by cooling down to room temperature. In a similar way, the MEMS die with backside metallization was subsequently soldered onto the silicon interlayer using Sn42Bi58 solder alloy with a melting point of 138 °C. After the assembled components cooled down to room temperature, standard wire bonding was conducted to electrically interconnect the MEMS die pads and the LCCC socket leads. Finally, vacuum pumping and parallel sealing were successively performed to encapsulate the wire-bonded MEMS die.

### 3.3. Analysis of Thermally Induced Packaging Effects

As mentioned in Section 2, a MEMS resonant accelerometer is a force-sensitive sensor that detects the resonant frequency variations of the resonant beam caused by the loaded inertial force to measure external acceleration. However, the scale factor and zero-bias stability of a MEMS resonant accelerometer are impacted by the thermally induced packaging effects. In particular, the thermo-mechanical stress formed during the die attachment process plays a dominant role as a source of packaging stress and adversely affects the performance of a MEMS resonant accelerometer [27,28].

Die attachment is a critical step in the packaging process of a MEMS resonant accelerometer, which is aimed at bonding the singulated MEMS die onto the specified area of the substrate or socket with DAF or solder alloy, thus establishing thermal, electrical, and mechanical connections. As shown in Figure 4, the die attachment process is consecutively implemented in two steps: first, the silicon interlayer is soldered onto the LCCC socket at an elevated temperature higher than the melting point of the SAC305 solder alloy, and second, the MEMS die is soldered onto the silicon interlayer at an elevated temperature higher than the melting point of the Sn42Bi58 solder alloy. At soldering temperature, the MEMS die is essentially stress-free with the minor correction of the intrinsic residual stress caused by the manufacturing process because the solder alloy is in the molten state and without the ability to transmit stress between the upper and lower structures. When the solder alloy cools down and solidifies gradually, the thermo-mechanical stress is generated in individual structures due to the deformation inconsistency among the structures of different CTEs. After cooling down to room temperature (or cooler), the packaged MEMS resonant accelerometer of dissimilar materials will experience large thermo-mechanical stress, since the CTE of the silicon MEMS die (2.6 ppm/°C) is much lower than that of the ceramic substrate and the solder layers (6.5~25 ppm/°C). Then, the stress is inevitably transmitted into the resonant beam through the fixing anchors, and consequently results in the frequency drift [8].

According to the analyses above, the thermo-mechanical stress can be reduced by matching the CTEs of dissimilar materials as far as possible, so as to minimize the thermally induced packaging effects on the frequency drift and improve the temperature stability of a MEMS resonant accelerometer. However, the packaging stress cannot be completely eliminated because of the ever-existing CTE mismatch.

**Figure 5:**
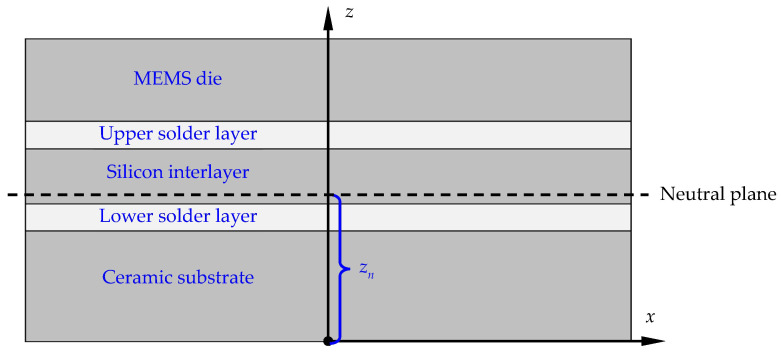
Schematic diagram of a five-layered laminate model for a packaged MEMS resonant accelerometer.

As shown in Figure 5, the packaged MEMS resonant accelerometer can be regarded as a five-layered laminate model, where all layers are perfectly bonded to each other without relative sliding. When it drops to room temperature from soldering temperature, bending of the five-layered laminate model takes place due to the CTE mismatch. Based on the plane hypothesis in classical beam bending theory, the upper part of the model is subjected to tensile stress, whereas the lower part is under compressive stress, or vice versa. The transition layer traversing through the model is defined as the neutral plane, where the normal stresses are zero. From the equilibrium equation describing that the net force of the normal stress on the cross section of the model remains zero, the location of the neutral plane can be derived as

(15) $$z_{\mathrm{np}}=\sum_{i=1}^{5}E_{i}S_{xi}/\sum_{i=1}^{5}E_{i}A_{i}$$

where *z* = *z*_np_ is the location of the neutral plane in the defined coordinate system shown in Figure 5; *E* and *A* are the Young’s modulus and cross-sectional area of each layer, respectively; *S_x_* is the static moment of each layer with respect to the x-axis; and the subscript *i* denotes the layer *i* in the model. Furthermore, in view of the fact that all layers are similar in the plane dimensions, Equation (15) can be approximatively rewritten as

(16) $$z_{\mathrm{np}}=\sum_{i=1}^{5}E_{i}t_{i}\left(z_{i-1}+\frac{t_{i}}{2}\right)/\sum_{i=1}^{5}E_{i}t_{i}$$

where $z_{i-1}=\sum_{j=1}^{i-1}t_{j}$ and *t* is the thickness of each layer in the model.

From Equation (16), it can be seen that the location of the neutral plane is correlated with the changes in the thickness of each layer. As the neutral plane approaches the resonant beam, the thermo-mechanical stress will be reduced dramatically, along with the frequency drift, which can be another way to improve the temperature stability of a MEMS resonant accelerometer.

## 4. Finite Element Simulations and Experimental Tests

### 4.1. Finite Element Modeling of MEMS Resonant Accelerometer

#### 4.1.1. Geometric Model and Material Properties

**Figure 6:**
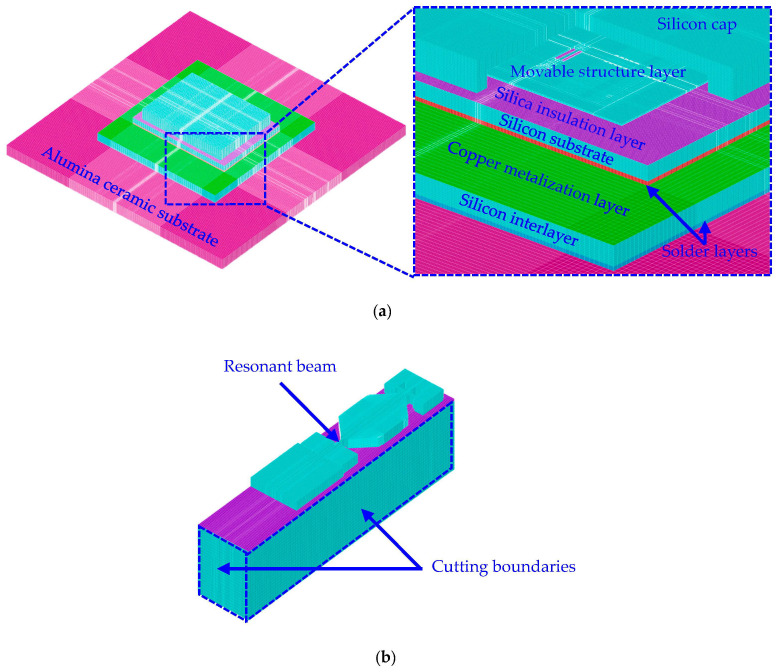
FEM models of the packaged MEMS resonant accelerometer: (**a**) global model; (**b**) local model separated from the global model.

As shown in Figure 6, 3D FEM models of the packaged MEMS resonant accelerometer were established with the element Solid 45 in the commercial software ANSYS 18.0. There are two separate models: the global model with relatively coarse mesh, including 4,171,617 elements and 4,376,598 nodes in total, as shown in Figure 6a, and the local model with more fine mesh of 1,011,504 elements and 1,054,162 nodes in total, as shown in Figure 6b. In the global model, the sizes of the silicon substrate and the movable structure layer are 7 mm × 5 mm × 380 μm and 4.5 mm × 3.5 mm × 50 μm, respectively. The sizes of the silicon cap and the vacuum cavity are 6 mm × 4.5 mm × 780 μm and 5 mm × 4 mm × 410 μm, respectively. The sizes of the silicon interlayer and the alumina ceramic substrate are 9 mm × 9 mm × 500 μm and 16.5 mm × 16.5 mm × 760 μm, respectively. The thicknesses of the copper metallization layer, the silica insulation layer, and the solder layers are 1 μm, 2 μm, and 100 μm, respectively. In the local model, the size of the resonant beam is 400 μm × 10 μm × 50 μm.

**Figure 7:**
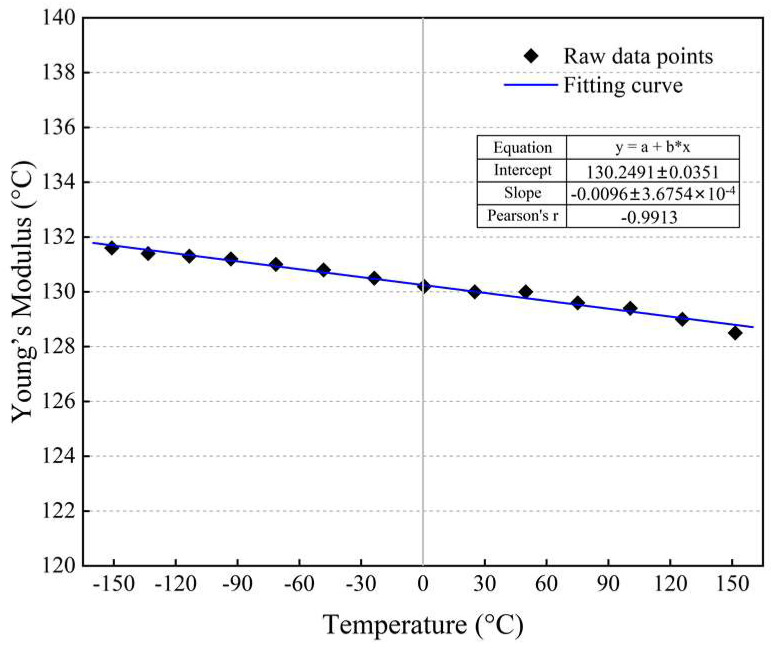
Temperature-dependent Young’s modulus of monocrystalline silicon.

Six dissimilar materials are involved in the FEM models, and the specific properties of these materials are listed in Table 1. In particular, monocrystalline silicon is modeled as an isotropic linearly elastic material with temperature-dependent properties, whose Young’s modulus decreases linearly with the temperature increasing from −150 °C to 150 °C, as shown in Figure 7 [29]. The lead-free solder alloy usually exhibits the viscoplastic characteristic at high temperatures, and for the sake of simplicity, it is assumed to be homogenous, isotropic, and linearly elastic. Similarly, alumina, silica, and copper are all modeled as isotropic linearly elastic materials.

#### 4.1.2. Thermo-Mechanical-Modal Simulation Procedures

For the sake of computing efficiency, the following assumptions were made throughout the thermo-mechanical-modal simulations: (1) all constituents in the FEM models are perfectly bonded to each other without defects. (2) There is no intrinsic residual stress in the models. (3) The temperature distribution in the models is uniform and is equal to the ambient temperature. The stress-free reference temperature for the MEMS die, the silicon interlayer, and the Sn42Bi58 solder layer was set as the melting point of the Sn42Bi58 solder alloy, about 138 °C. The stress-free reference temperature for the SAC305 solder layer and the ceramic substrate was set as the melting point of the SAC305 solder alloy, about 218 °C. The simulations were implemented within the temperature range of 0 °C to 90 °C, and the solutions were obtained for every 15 °C. In addition, the fixed constraints were applied at the sidewalls of the ceramic substrate to prevent rigid body motion.

The thermo-mechanical-modal simulations were indirectly carried out using the global-local technique, which can reduce the computing cost without sacrificing accuracy [17,32]. Firstly, the global model was cooled down from the stress-free temperature to the given temperature, where it was necessary to calculate the resonant frequency. The deformation and stress distribution of the MEMS resonant accelerometer were captured through the thermo-mechanical simulation. Then, the displacement interpolation boundary conditions extracted from the global model were applied to the local model cutting boundaries, where the local model was separated from the global model. Moreover, the identical given temperature load was applied to the local model, and more accurate stress results were calculated through the thermo-mechanical simulation. After that, the stress distribution was transferred to the prestressed modal simulation. Finally, the frequency drift of the MEMS resonant accelerometer induced by temperature variations was obtained.

### 4.2. Temperature Tests for MEMS Resonant Accelerometer

**Figure 8:**
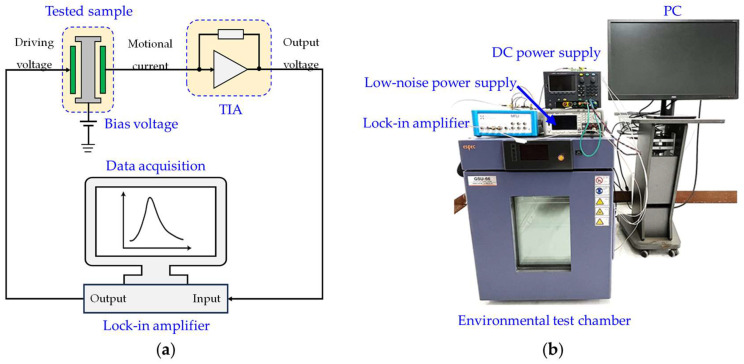
Measuring system of the frequency response characteristic of the MEMS resonant accelerometer: (**a**) electrical control loop; (**b**) experimental setup of the temperature test.

Moreover, the changes in the resonant frequency of the MEMS resonant accelerometer with temperature variations were also measured experimentally. The schematic diagram of measuring the frequency response characteristic is shown in Figure 8a, and the experimental setup of the temperature test is illustrated in Figure 8b. The tested sample was placed in an environmental test chamber to simulate the ambient temperature variation, and a bias voltage was applied on the tested sample through a low-noise power supply to facilitate the actuation. The tested sample was capacitively driven into linear operation through the AC driving signal derived from a lock-in amplifier (Zurich Instruments MFLI, Zurich, Switzerland). A trans-impedance amplifier (TIA) was powered by DC power supply to convert the output motional current from the tested sample into a voltage, which was fed back into the lock-in amplifier for a closed-loop implementation. The frequency response characteristics of the tested samples were tracked by the phase-locked loop (PLL) feature of the MFLI system. The resonant frequency of the MEMS resonant accelerometer was measured for every 15 °C, and the measurements at each temperature point were repeated from 0 °C to 90 °C.

## 5. Results and Discussions

### 5.1. Thermo-Mechanical Simulation Results

#### 5.1.1. Thermal Deformation of MEMS Resonant Accelerometer

**Figure 9:**
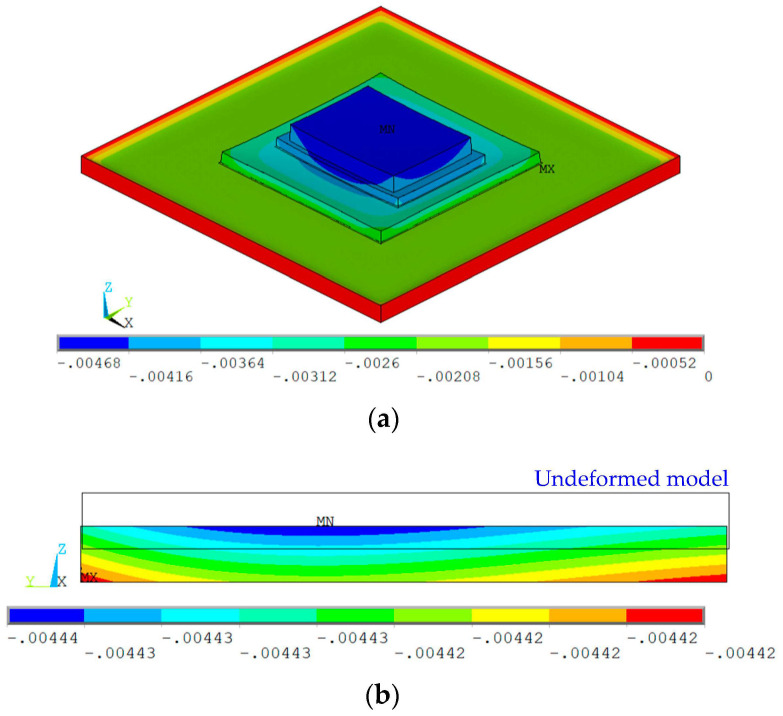
Thermal deformation obtained through the thermo-mechanical simulations: (**a**) deformation of the packaged MEMS resonant accelerometer at room temperature; (**b**) deformation of the resonant beam at room temperature.

Figure 9a shows the thermal deformation of the packaged MEMS resonant accelerometer after cooling down to room temperature from the stress-free temperature, which was derived from the thermo-mechanical simulation of the global model. When the ambient temperature decreases, the silicon interlayer and the MEMS die tend to shrink more than the ceramic substrate, which cannot deform freely because of the mechanical constraints, thus leading to the deformation of the assembled component in an upwards concave or smile shape.

Figure 9b shows the thermal deformation of the resonant beam captured through the thermo-mechanical simulation of the local model as the ambient temperature dropped to room temperature. Due to the warpage of the MEMS die, the resonant beam shifts downwards and bends upwards slightly in comparison to the undeformed model. The maximum warpage was calculated to be about 8 × 10^−3^ μm, which was derived from the z-axis coordinate of the highest point subtracting that of the lowest points on the top surface of the resonant beam. In spite of the minor deformation, this causes a change in the effective stiffness of the resonant beam and, consequently, leads to the frequency drift.

#### 5.1.2. Thermo-Mechanical Stress of MEMS Resonant Accelerometer

**Figure 10:**
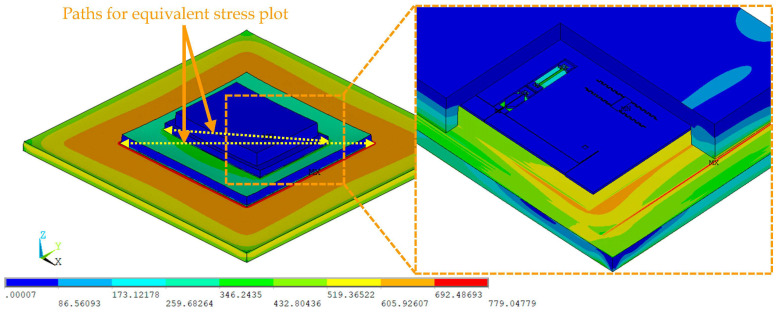
Equivalent stress distribution within the packaged MEMS resonant accelerometer at room temperature.

Figure 10 shows the equivalent stress distribution within the packaged MEMS resonant accelerometer after cooling down to room temperature from the stress-free temperature. The maximum equivalent stress is approximately 779.05 MPa, which is located at the SAC305 solder layer between the silicon interlayer and the ceramic substrate. This is attributed to the fact that the higher CTE of the SAC305 solder alloy relative to the silicon interlayer and the ceramic substrate causes large thermo-mechanical stress. With regard to the MEMS die, the thermo-mechanical stress is mainly distributed around the interface between the silicon cap and the silica insulation layer on the silicon substrate because of the CTE mismatch. In addition, due to the fact that the movable structure layer is isolated from the silicon substrate with the supporting structures, the thermo-mechanical stress within the movable structure layer is quite low.

**Figure 11:**
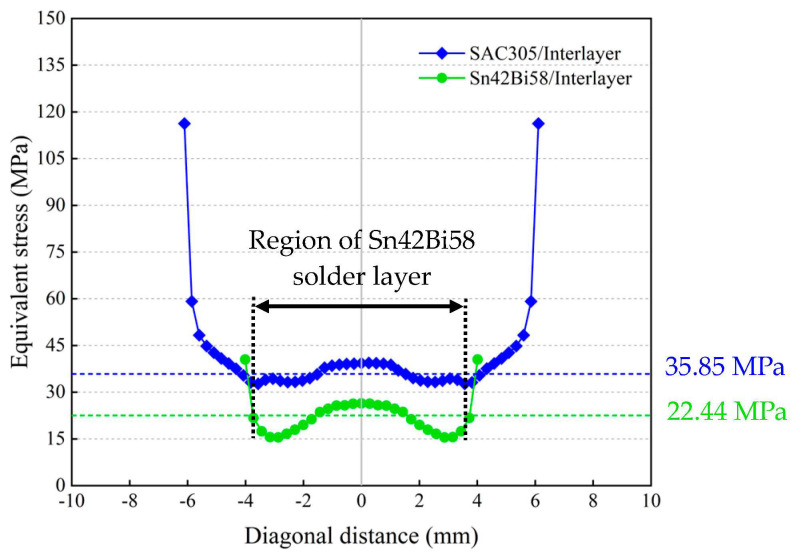
Equivalent stress plots along the diagonals at the bonding interfaces between the silicon interlayer and the solder layers at room temperature.

In order to assess the stress-buffering effect of the silicon interlayer, the equivalent stresses were extracted along the diagonals (dotted lines as illustrated in Figure 10) on the bonding interfaces between the silicon interlayer and the solder layers, as plotted in Figure 11. This shows that the equivalent stresses at the bonding interface between the silicon interlayer and the Sn42Bi58 solder layer are much lower compared to those at the bonding interface between the silicon interlayer and the SAC305 solder layer. The average equivalent stress within the region of the Sn42Bi58 solder layer drops to 22.44 MPa from 35.85 MPa, which is a reduction of 37.41%. This reveals that the silicon interlayer can effectively relax the thermo-mechanical stress transmitted from the ceramic substrate into the MEMS die.

**Figure 12:**
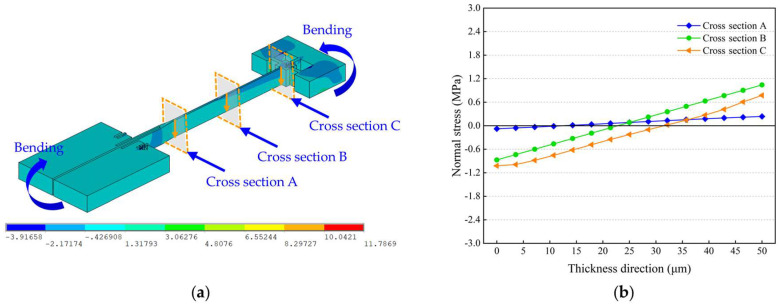
Thermo-mechanical stress within the resonant beam at room temperature: (**a**) axial stress distribution along the length direction of the resonant beam; (**b**) normal stresses on the cross sections along the thickness direction of the resonant beam.

Figure 12a shows the axial stress distribution along the length direction of the resonant beam at room temperature. It can be seen that the upper part of the resonant beam is subjected to compressive stress, whereas the lower part is under tensile stress because of the bending deformation, as indicated in Figure 9. The detailed normal stresses on the cross sections were extracted along the thickness direction of the resonant beam, as illustrated in Figure 12a. As shown in Figure 12a, Cross sections A and C are located at the root of the turning fork and the fixed end of the resonant beam, respectively, and Cross section B is in the middle between Cross sections A and C. Figure 12b shows that the normal stresses on the cross sections vary linearly in general along the thickness direction of the resonant beam. From the top to bottom of the resonant beam, the compressive stress decreases from −0.08 MPa to 0 MPa, and the tensile stress increases from 0 MPa to 0.24 MPa on Cross section A. On Cross section B, the compressive stress decreases from −0.87 MPa to 0 MPa and the tensile stress increases from 0 MPa to 1.04 MPa. On Cross section C, the compressive stress decreases from −1.02 MPa to 0 MPa and the tensile stress increases from 0 MPa to 0.77 MPa. This corresponds to the beam bending characteristics, which means that the normal stress at the location far from the neutral plane is much higher.

**Figure 13:**
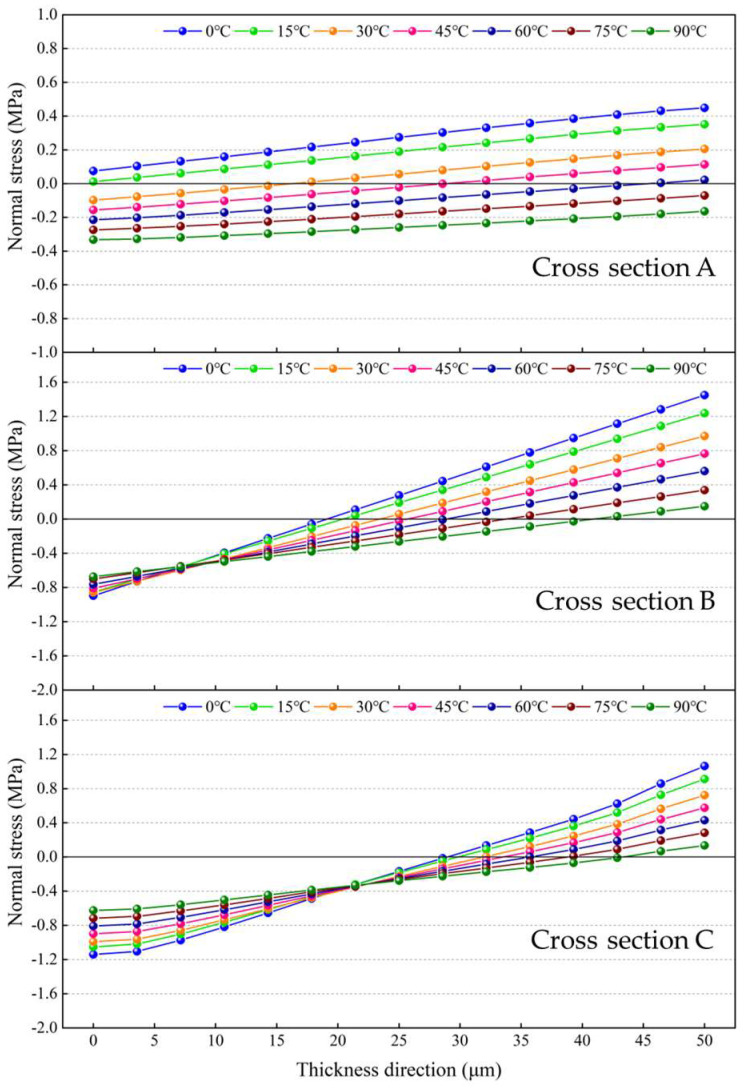
Normal stresses on the cross sections of the resonant beam over 0 °C to 90 °C.

Figure 13 shows the normal stresses on the cross sections of the resonant beam at different ambient temperatures. As the ambient temperature rises, the dominant normal stress on Cross section A changes from tensile stress to compressive stress, and the closer to the bottom surface of the resonant beam, the more significant the change in the compressive stress is. With regard to Cross sections B and C, the normal stress decreases with increasing temperature, and the closer to the bottom surface of the resonant beam, the more significant the decline in the normal stress is. When the ambient temperature rises to 90 °C from 0 °C, the compressive stress gradually becomes the dominant normal stress within the resonant beam instead of the tensile stress. The maximum declines in the tensile stresses on Cross sections B and C were calculated to be about 1.27 MPa and 1.51 MPa, respectively. The decline in the tensile stress and the increment in the compressive stress cause the effective stiffness reduction of the resonant beam and, consequently, lead to the reduction in the resonant frequency, as indicated by Equation (6).

### 5.2. Prestressed Modal Simulation Results

**Figure 14:**
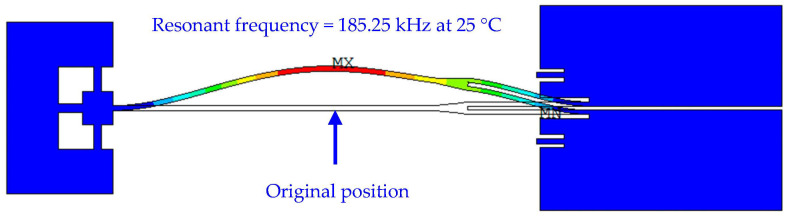
Simulated first mode shape of the resonant beam at room temperature.

After the thermo-mechanical simulations, prestressed modal simulations were conducted to obtain the mode shapes and resonant frequencies of the resonant beam. As shown in Figure 14, the resonant beam with the first-order mode shape is curved in the shape of a bow at room temperature and the displacement vector is perpendicular to the detection direction of external acceleration, which is along the axial direction of the resonant beam. The simulated resonant frequency of the resonant beam is approximately 185.25 kHz at room temperature.

**Figure 15:**
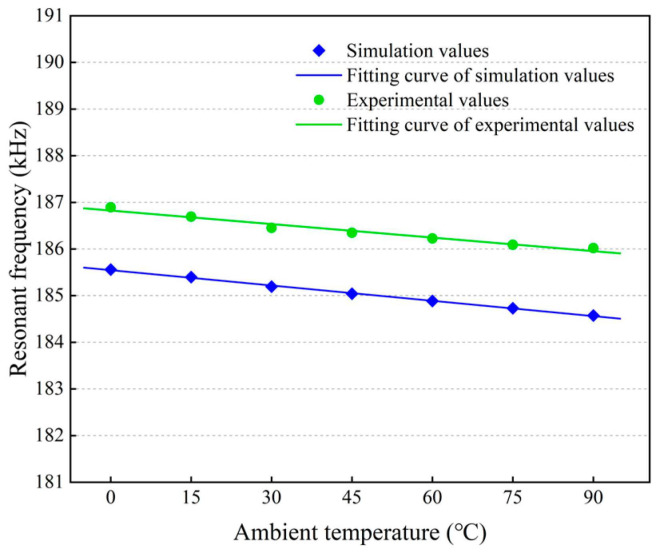
Simulated and measured resonant frequencies of the resonant beam at different temperatures.

Figure 15 plots the resonant frequencies of the resonant beam at different temperatures obtained through the prestressed modal simulations and the temperature tests. The temperature coefficient of resonant frequency is obtained via

(17) $$k=\frac{f_{T}-f_{T_{0}}}{\left(T-T_{0}\right)f_{T_{0}}}$$

where *f_T_* is the resonant frequency at the temperature *T*.

When the ambient temperature rises to 90 °C from 0 °C, the simulated resonant frequency decreases linearly from 185.56 kHz to 184.57 kHz, and the measured resonant frequency decreases linearly from 186.89 kHz to 186.02 kHz. This is due to the decreasing tensile stress and the dominant compressive stress within the resonant beam, as mentioned above. The simulated temperature coefficient was calculated to be about −59.28 ppm/°C and the measured temperature coefficient was calculated to be about −51.74 ppm/°C. As indicated in Section 2, the frequency drift correlated with the thermo−mechanical stress formed in chip-level packaging is comparable to that correlated with the Young’s modulus sensitivity to temperature. Furthermore, this shows that the simulated resonant frequencies are less than the measured values. This could be attributed to the fact that the idealized FEM model is assumed to be residual stress-free and without regard to the actual manufacturing deviations of the device. The maximum relative error of the resonant frequency is less than 1%, and the relative error of the temperature coefficient is less than 15%, which verifies the availability and accuracy of the finite element simulations.

### 5.3. Influences of Packaging Structure Parameters on the Frequency Drift of MEMS Resonant Accelerometer

#### 5.3.1. Influences of the Silicon Interlayer Thickness

**Figure 16:**
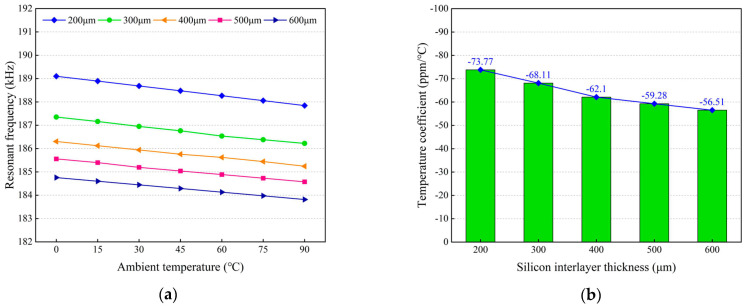
Influences of the silicon interlayer thickness on the frequency response characteristic of the resonant beam: (**a**) simulated resonant frequencies of the resonant beam; (**b**) simulated temperature coefficients of resonant frequency.

Firstly, the influences of the silicon interlayer thickness on the frequency drift of the MEMS resonant accelerometer were investigated. Figure 16a shows the resonant frequencies of the resonant beam with different silicon interlayer thicknesses. It can be seen that the resonant frequencies decrease as the silicon interlayer thickness increases. Furthermore, the resonant frequencies decrease linearly with the temperature increasing from 0 °C to 90 °C, which means that the temperature coefficients are negative for all cases. Figure 16b shows the simulated temperature coefficients of resonant frequency with different silicon interlayer thicknesses. The magnitude of the temperature coefficient decreases with the increase in the silicon interlayer thickness, but the declining amplitude gradually slows down. When the silicon interlayer thickness is increased from 200 μm to 600 μm, the simulated temperature coefficient decreases from 73.77 ppm/°C to 56.51 ppm/°C.

**Figure 17:**
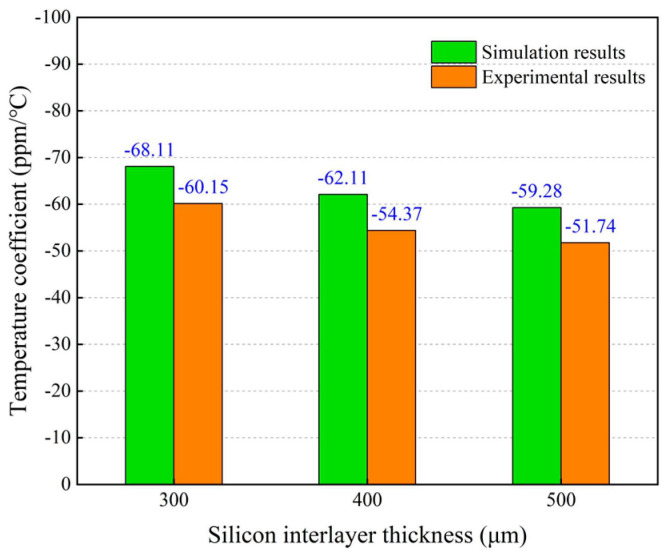
Comparison of the simulated and measured temperature coefficients of resonant frequency with different silicon interlayer thicknesses.

By means of the experimental setup shown in Figure 8, the resonant frequency-temperature curves of the MEMS resonant accelerometer with different silicon interlayer thicknesses were measured, and the temperature coefficients of resonant frequency were calculated from these curves. The comparison of the simulated and measured temperature coefficients with different silicon interlayer thicknesses is illustrated in Figure 17. It reveals that the variation trend of the simulated temperature coefficients is in good accordance with that of the experimental results. When the silicon interlayer thickness is increased from 300 μm to 500 μm, the measured temperature coefficient decreases from 60.15 ppm/°C to 51.74 ppm/°C. The maximum relative error between the measured and simulated temperature coefficients is less than 15%.

**Figure 18:**
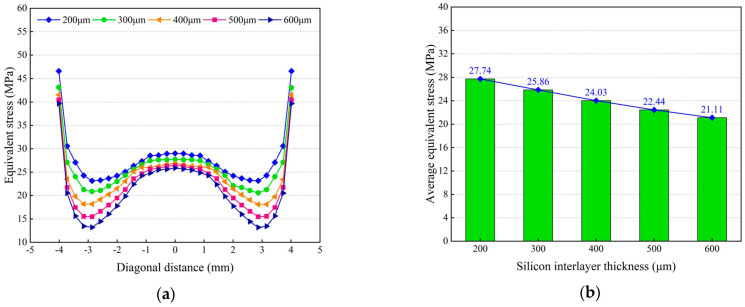
Influences of the silicon interlayer thickness on stress-buffering effect: (**a**) equivalent stress plots along the diagonals on the bonding interface between the silicon interlayer and the Sn42Bi58 solder layer at room temperature; (**b**) average values of the equivalent stresses.

**Figure 19:**
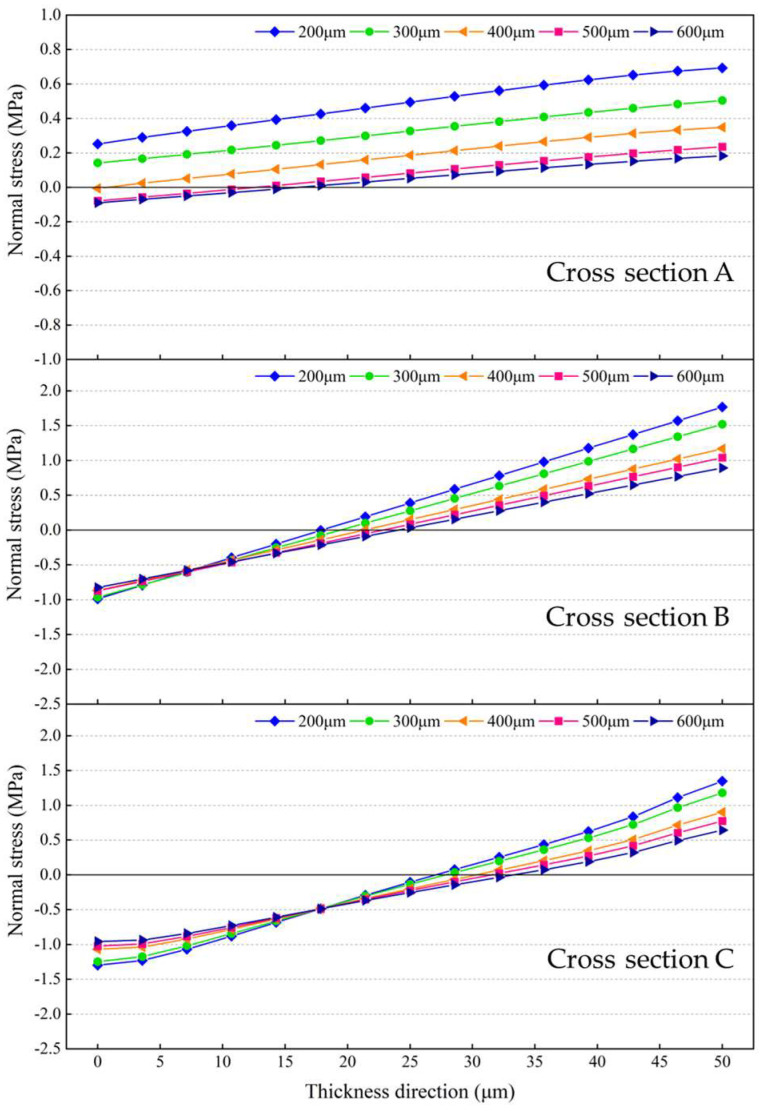
Normal stresses on the cross sections of the resonant beam with different silicon interlayer thicknesses at room temperature.

Figure 18a plots the equivalent stresses extracted along the diagonal at the bonding interface between the silicon interlayer and the Sn42Bi58 solder layer at room temperature. It shows that the equivalent stresses decrease as the silicon interlayer thickness increases. When the silicon interlayer thickness is increased from 200 μm to 600 μm, the average equivalent stress decreases from 27.74 MPa to 21.11 MPa, as shown in Figure 18b. Figure 19 plots the normal stresses on the cross sections of the resonant beam as illustrated in Figure 12a at room temperature. When the silicon interlayer thickness is less than 400 μm, the dominant normal stress on Cross section A is tensile stress and it decreases with the increase in the silicon interlayer thickness. As the silicon interlayer thickness is increased than 400 μm, the upper part of Cross section A is subjected to compressive stress, whereas the lower part is under tensile stress. With regard to Cross sections B and C, the magnitudes of the compressive and tensile stresses both decrease as the silicon interlayer thickness increases. It can be seen that the declining amplitude of the tensile stress is more significant. When the silicon interlayer thickness is increased from 200 μm to 600 μm, the tensile stresses decrease from 0.69 MPa to 0.18 MPa on Cross section A, from 1.77 MPa to 0.89 MPa on Cross section B, and from 1.35 MPa to 0.65 MPa on Cross section C. This indicates that increasing the silicon interlayer thickness can improve the stress-buffering effect and significantly minimize the thermo-mechanical stress within the resonant beam.

**Figure 20:**
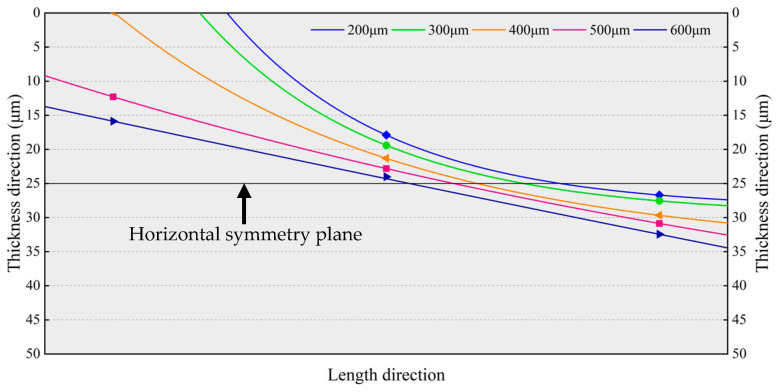
Neutral planes of the resonant beams with different silicon interlayer thicknesses.

Figure 20 plots the neutral planes of the resonant beams depicted by connecting the points shown in Figure 19, where the normal stresses are zero. The normal stress below the neutral plane is tensile stress, whereas the normal stress above the neutral plane is compressive stress. As the silicon interlayer thickness increases, the center points of the neutral plane and horizontal symmetry plane of the resonant beam gradually coincide. The resonant beam is approximatively divided into two centrosymmetric parts by the neutral plane. Thus, the effects of the compressive and tensile stresses on the effective stiffness of the resonant beam will cancel each other out to some extent, and the frequency drift of the MEMS resonant accelerometer can be minimized as well.

#### 5.3.2. Influences of the Thickness of Sn42Bi58 Solder Layer

**Figure 21:**
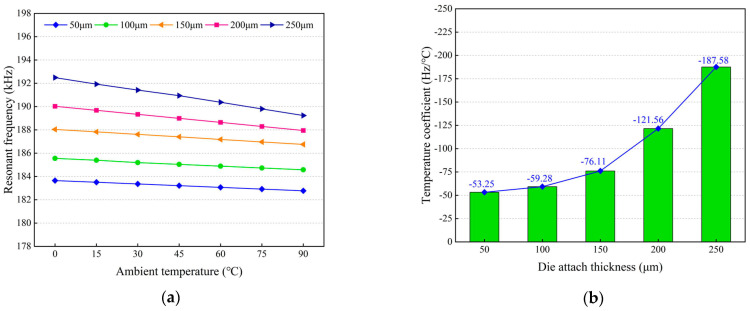
Influences of the thickness of the Sn42Bi58 solder layer on the frequency response characteristic of the resonant beam: (**a**) simulated resonant frequencies of the resonant beam; (**b**) simulated temperature coefficients of resonant frequency.

Next, the frequency drift of the MEMS resonant accelerometer with different thicknesses of the Sn42Bi58 solder layer were analyzed. Figure 21a shows that the resonant frequencies of the resonant beam increase with the increase in the thickness of the Sn42Bi58 solder layer and the resonant frequencies decrease linearly with the temperature increasing from 0 °C to 90 °C for all cases. Furthermore Figure 21b shows that the magnitude of the simulated temperature coefficient increases as the thickness of the Sn42Bi58 solder layer increases and the increasing amplitude is gradually increased. When the thickness of the Sn42Bi58 solder layer is increased from 50 μm to 250 μm, the simulated temperature coefficient increases from 53.25 ppm/°C to 187.58 ppm/°C.

**Figure 22:**
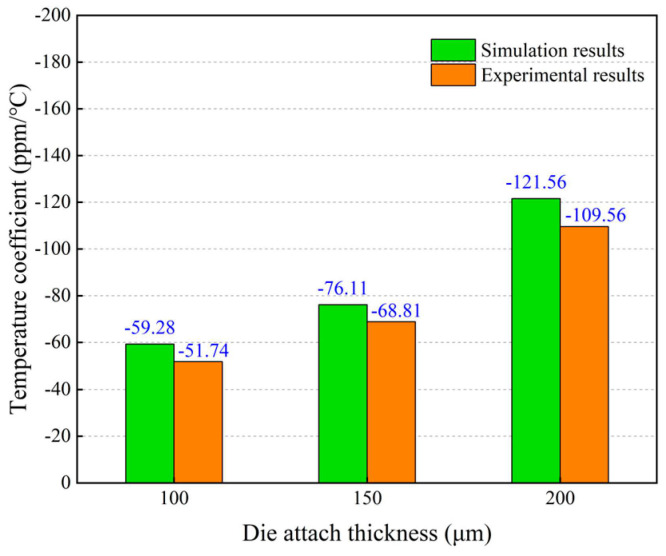
Comparison of the simulated and measured temperature coefficients of resonant frequency with different thicknesses of Sn42Bi58 the solder layer.

Figure 22 compares the simulated and measured temperature coefficients with different thicknesses of the Sn42Bi58 solder layer. It reveals that the variation trend of the simulated temperature coefficients is in good accordance with that of the experimental results. When the thickness of the Sn42Bi58 solder layer is increased from 50 μm to 250 μm, the measured temperature coefficient increases from 51.74 ppm/°C to 109.56 ppm/°C. The maximum relative error between the measured and simulated temperature coefficients is less than 15%.

**Figure 23:**
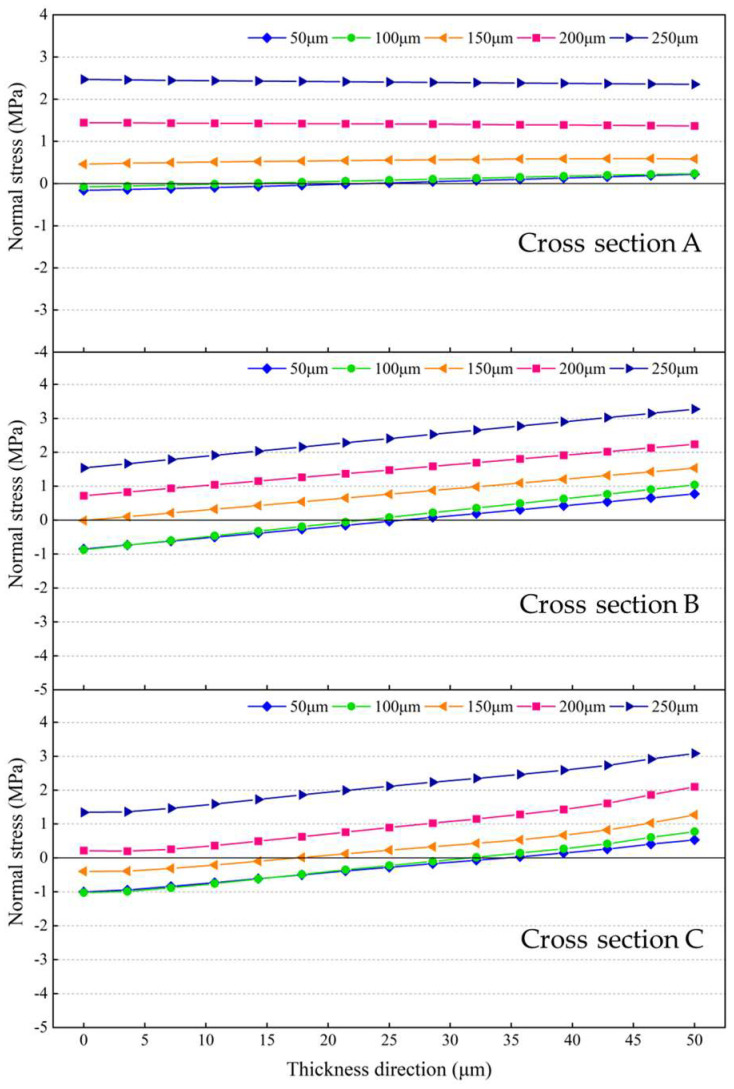
Normal stresses on the cross sections of the resonant beam with different thicknesses of the Sn42Bi58 solder layer at room temperature.

Figure 23 plots the normal stresses on the cross sections of the resonant beam as illustrated in Figure 12a with different thicknesses of the Sn42Bi58 solder layer at room temperature. It can be seen that the tensile stresses become the dominant normal stresses on the cross sections and gradually increase with the increase in the thickness of the Sn42Bi58 solder layer. When the thickness of the Sn42Bi58 solder layer is less than 150 μm, the upper parts of the cross sections are subjected to compressive stress, whereas the lower parts are under tensile stress. This indicates that reducing the thickness of the Sn42Bi58 solder layer is beneficial to minimize thermo-mechanical stress within the resonant beam.

**Figure 24:**
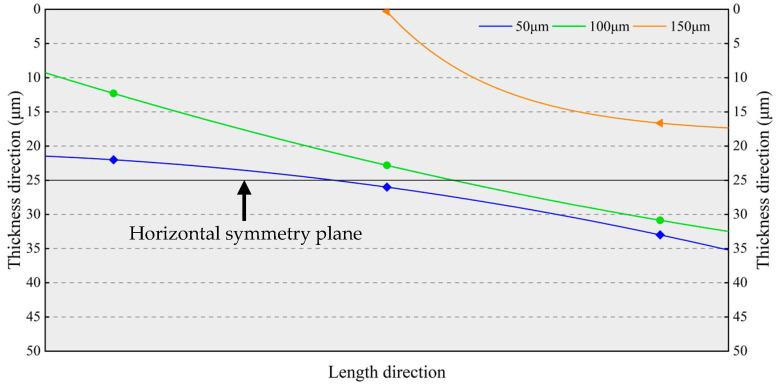
Neutral planes of the resonant beams with different thicknesses of the Sn42Bi58 solder layer.

Figure 24 plots the neutral planes of the resonant beams depicted by connecting the points shown in Figure 23, where the normal stresses are zero. The normal stress below the neutral plane is tensile stress, whereas the normal stress above the neutral plane is compressive stress. When the thickness of the Sn42Bi58 solder layer is increased to more than 150 μm, the neutral plane is out of the resonant beam, which is subjected to the tensile stress. As the thickness of the Sn42Bi58 solder layer decreases, the resonant beam is approximatively divided into two centrosymmetric parts by the neutral plane. Due to the mutual compensation for the effective stiffness variations of the resonant beam resulting from the compressive and tensile stresses, the frequency drift of the MEMS resonant accelerometer will be minimized.

#### 5.3.3. Comparison of the Influences of the Packaging Structure Parameters

Furthermore, the influences of the silicon interlayer thickness and the thickness of the Sn42Bi58 solder layer on the frequency drift of the MEMS resonant accelerometer were compared through the Taguchi method, which is widely used for orthogonal experimental designs. In the experimental design analyses, the signal-to-noise (S/N) ratios were derived from the simulated temperature coefficients of resonant frequency. The larger the value of the S/N ratio, the more significant the influence on the frequency drift. As listed in Table 2, a L_9_(3^2^) orthogonal array with two factors and three levels was established for the experimental design analyses. In view of the purpose of minimizing the frequency drift of the MEMS resonant accelerometer, the smaller-the-better type of S/N ratios were calculated via

(18) $$S/N=-10\times \log\left(\frac{1}{n}\sum_{i=1}^{n}k_{i}^{2}\right)$$

where *n* is the number of experiments with the same design parameter combination, which was taken as one due to the fact that the experimental design analyses were conducted through finite element simulations, and *k_i_* is the simulated temperature coefficient of resonant frequency for each design parameter combination.

**Figure 25:**
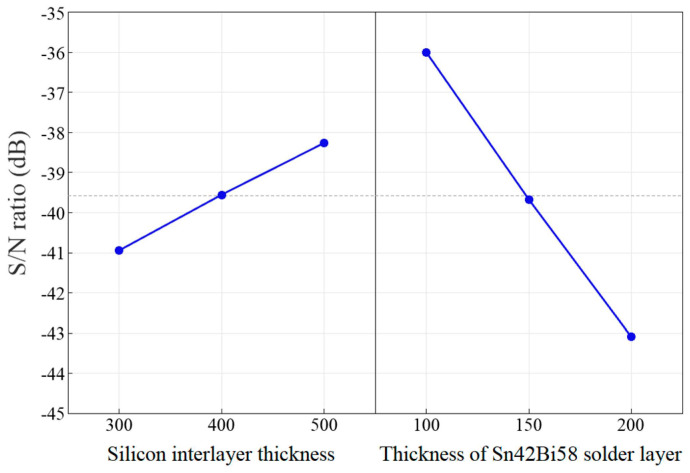
Average S/N ratio responses of the two factors.

The temperature coefficients of resonant frequency and S/N ratios for all design parameter combinations are listed in Table 3. The average S/N ratio responses for each level of the two factors were calculated as shown in Figure 25. It reveals that the thickness of the Sn42Bi58 solder layer has a more significant influence on the frequency drift compared to the silicon interlayer thickness. This indicates that reducing the thickness of the Sn42Bi58 solder layer is quite an effective way to minimize the thermally induced packaging effects on the frequency drift of the MEMS resonant accelerometer. On the other hand, the thickness of the Sn42Bi58 solder layer should not be too thin to ensure sufficient soldering strength.

## 6. Conclusions

In this paper, the thermal effects on the frequency drift of a MEMS resonant accelerometer were systematically analyzed. Our analysis indicates that the thermo-mechanical stress formed in chip-level packaging plays a dominant role in the frequency drift of the MEMS resonant accelerometer, apart from the Young’s modulus of monocrystalline silicon. An LCCC package with a stress-buffering layer was proposed for the MEMS resonant accelerometer, and the influences of packaging structure parameters on the frequency drift were investigated using finite element simulations and verified through temperature tests. Due to the CTE mismatch between dissimilar materials, the MEMS resonant accelerometer package deforms in a concave upwards shape, and the bending stress within the resonant beam leads to a change in the effective stiffness, which consequently causes the frequency drift of the MEMS resonant accelerometer. As the ambient temperature increases, the resonant frequency of the resonant beam decreases linearly. This reveals that increasing the silicon interlayer thickness can improve the stress-buffering effect and reduce the thermo-mechanical stress within the resonant beam, along with the frequency drift. Moreover, in comparison to the silicon interlayer thickness, reducing the thickness of the Sn42Bi58 solder layer can more effectively minimize the frequency drift of the MEMS resonant accelerometer. The findings showed that as the neutral plane approaches the horizontal symmetry plane of the resonant beam, the effects of the compressive and tensile stresses on the effective stiffness of the resonant beam will cancel each other out to some extent, which consequently can reduce the frequency drift of the MEMS resonant accelerometer. This paper explores an effective way to minimize the thermally induced packaging effects on the frequency drift and improve the temperature stability of a MEMS resonant accelerometer.

## Figures and Tables

**Table 1:** Material properties of the FEM models.

Material	Young’s Modulus (GPa)	Poisson’s Ratio	CTE (ppm/°C)	Density (g/cm^3^)
Si [29]	Figure 8	0.28	2.60	2.33
SiO_2_	70	0.20	0.35	2.50
Cu	129	0.35	16.90	8.92
Al_2_O_3_	310	0.22	7.00	3.97
SAC305 [30]	55	0.36	17.00	7.30
Sn42Bi58 [31]	38	0.35	15.00	8.70

**Table 2:** Levels of the two factors.

Factor	Level 1	Level 2	Level 3
Silicon interlayer thickness (μm)	300	400	500
Thickness of Sn42Bi58 solder layer (μm)	100	150	200

**Table 3:** Experimental design analysis results.

Combination	Silicon Interlayer Thickness (μm)	Thickness of Sn42Bi58 Solder Layer (μm)	Temperature Coefficient of Resonant Frequency (ppm/°C)	S/N Ratio (dB)
A	300	100	−68.11	−36.66
B	300	150	−122.62	−41.77
C	300	200	−165.80	−44.39
D	400	100	−62.11	−35.86
E	400	150	−95.51	−39.60
F	400	200	−144.41	−43.19
G	500	100	−59.28	−35.46
H	500	150	−76.11	−37.63
I	500	200	−121.56	−41.70

## Data Availability

Data will be available on request.
